# SOLETE, a 15-month long holistic dataset including: Meteorology, co-located wind and solar PV power from

**DOI:** 10.1016/j.dib.2022.108046

**Published:** 2022-03-13

**Authors:** Daniel Vazquez Pombo, Oliver Gehrke, Henrik W. Bindner

**Affiliations:** a Department of Electrical Engineering, Technical University of Denmark (DTU), Frederikborsvej 399, Roskilde 4000, Denmark; b R&D Strategic Development, Vattenfall AB, Evenemangsgatan 13C, Solna 169 56, Sweden

**Keywords:** Wind power, Solar power, Irradiance, Wind speed, Wind direction, Humidity, Photovoltaic, Pressure

## Abstract

The aim of the SOLETE dataset is to support researchers in the meteorological, solar and wind power forecasting fields. Particularly, co-located wind and solar installations have gained relevance due to the rise of hybrid power plants and systems. The dataset has been recorded in SYSLAB, a laboratory for distributed energy resources located in Denmark. A meteorological station, an 11 kW wind turbine and a 10 kW PV array have been used to record measurements, transferred to a central server. The dataset includes 15 months of measurements from the 1st June 2018 to 1st September 2019 covering: Timestamp, air temperature, relative humidity, pressure, wind speed, wind direction, global horizontal irradiance, plane of array irradiance, and active power recorded from both the wind turbine and the PV inverter. The data was recorded at 1 Hz sampling rate and averaged over 5 min and hourly intervals. In addition, there are three Python source code files accompanying the data file. *RunMe.py* is a code example for importing the data. *MLForecasting.py* is a self-contained example on how to use the data to build physics-informed machine learning models for solar PV power forecasting. *Functions.py* contains utility functions used by the other two.

## Specifications Table

**Table:** 

Subject	Energy Engineering and Power Technology
Specific subject area	Meteorological and active power recordings suitable for time-series forecasting, from a site including a 11 kW wind turbine (WT) and a 10 kW PV array
Type of data	Table (.hdf5), Script (.py), Figure (.png).
How the data were acquired	Pyranometer (SolData 243SPC), Humidity and Temperature Probe (Vaisala Oyj, HMP155), Wind Vane (WZOOP), WindSensor P2546AOPR Cup Anemometer, WS600-UMB Smart Weather Sensor, Gaia WT, Schu¨co PV pannels, SMA SunnyTripower 10000TL.
Data format	Interpreted, raw data has been translated into SI units and down-sampled.
Description of data collection	The raw data was recorded at a 1 Hz sample rate, timestamped with respect to Unix time, interpreted as SI units, transmitted to a central logger, and stored in csv files along with the values from other devices present in SYSLAB. To build the dataset, these files were imported, only selecting the interesting metrics, and averaged to obtain 5 min and hourly resolution.
Data source location	• Institution: Risø DTU National Laboratory for Sustainable Energy • City: Roskilde • Country: Denmark • Latitude and longitude: 55.6867, 12.0985
Data accessibil- ity	Repository name: DTU Data Data identification number: https://doi.org/10.11583/DTU.17040767 Direct URL to data: https://data.dtu.dk/articles/dataset/The_SOLETE_dataset/17040767 The SOLETE dataset/17,040,767 Corresponding Reference: [1] Repository name: DTU Data Software release number: https://doi.org/10.11583/DTU.17040626 Direct URL to release: https://github.com/DVPombo/SOLETE The SOLETE platform/17,040,626 Corresponding Reference: [2]
Related research articles	[3] D. V. Pombo, H. W. Bindner, S. V. Spataru, P. E. Sørensen, & P. Bacher, Increasing the Accuracy of Hourly Multi-Output Solar Power Forecast with Physics-Informed Machine Learning, Sensors 22 (3) (2022) 749. [4] D. V. Pombo, P. Bacher, C. Ziras, H. W. Bindner, S. V. Spataru, & P. E. Sørensen, Benchmarking Physics-Informed Machine Learning-based Short Term PV-Power Forecasting Tools, Under Review. [5] D. V. Pombo, T. G¨oc¸men, K. Das, & P. Sørensen, Multi-Horizon DataDriven Wind Power Forecast: From Nowcast to 2 Days-Ahead. In 2021 International Conference on Smart Energy Systems and Technologies (SEST) (pp. 1–6). IEEE. (2021, September).

## Value of the Data

This data is useful for either meteorological, renewable energy or big data studies as it is a collection of recordings from both atmospheric conditions and energy production.

• The resolution and length of SOLETE makes it particularly useful for the forecasting community in both the meteorological and power fields. Specially for those working in machine learning (ML) and big data fields.
• The dataset was originally developed for one to three days-ahead forecasting of solar and wind power using data-driven methods such as ML. The ongoing discussion of the renewable community regarding forecasting meteorological metrics or directly power requires honest comparisons. This dataset and the related publications can serve as a baseline.
• The dataset is complemented by a Git repository [2] including three Python scripts employing only Open Access libraries. The first script simply imports the data, while the second showcases the methodology discussed in [3] and [4] to build physics-informed ML-models for solar power forecasting. This resource is particularly useful for students and researchers first starting in the machine learning-based forecasting field.

## 1 Data Description

The dataset [1] and its related repository [2] contain five files: *SOLETE Pombo* 60 min*.h5, SOLETE Pombo* 5 min*.h5, RunMe.py, MLForecasting.py*, and *Functions.py*.

• *Solete Pombo* 5 min*.h5* and *SOLETE Pombo* 60 min*.h5*: Correspond to the dataset files containing the recordings from SYSLAB. These are 15 months of measurements from the 1st June 2018 to 1st September 2019. Hence a total of 10,969 and 131,617 samples for hourly and 5 min, respectively covering 10 metrics: Unix timestamp, air temperature [°C], relative humidity [%], wind speed [m/s], wind direction [deg], global horizontal irradiance [kW/m^2^], plane of array irradiance [kW/m^2^], pressure [mbar], and active power [kW] recorded from both the WT and the PV inverter. Note that the timestamps correspond to UTC time in the format “year-month-day hour:minute second” or “yyyy-mm-dd hh:MM:ss”.
• *RunMe.py* This is a simple Python script meant to help users to start their work with the dataset. It imports the h5 file into the DATA variable as a pandas DataFrame [6]. After execution, the dictionaries PVinfo and WTinfo contain relevant data regarding the PV system and the Gaia turbine corresponding to that of Tables 1 and 2. Dependencies: Python 3.8.10, and pandas 1.2.4.

**Table 1:** Description of PVinfo contents.

Item	Content Description
Type	PV cell type (e.g. mono- or polycristaline
Az	Azimuth, inclination [deg]
Estc	Reference irradiance under standard conditions (STC) [W/m*^s^*]
Tstc	Reference temperature [*^circ^*^ˆ^ C]
Pmp stc	Peak power measured under STC [W]
ganma mp	Normalized temperature coefficient of peak power [1/K]
Ns	Number of panels connected in series
Np	Number of panels connected in parallel
a and b	Module material and construction parameters
D T	Estimated temperature difference between module and cell
eff_P and eff_%	Lookup table representing the efficiency curve of the array's inverter as W and %

**Table 2:** Description of WTinfo contents.

Item	Content Description
Type	Generator type
Mode	Wind following mode (constant value)
Pn	Nominal Power [kW]
Vn	Operating Voltage [V]
CWs and CP	Lookup table representing the turbine's power curve as m/*sec* vs kW
Cin and Cout	Cut-in and cut-out wind speeds [m/s]
HH	Hub height [m]
D	Rotor diameter [m]
SA	Swept area [m^2^]
B	Number of blades
• *MLForecasting.py* This file is directly related to the dataset's twin publications [3,4]. It is a self contained example of how to build a physics informed solar power forecaster based on different off-the-shelf ML algorithms. First, it imports the dataset, and expands it according to the methodology proposed in both papers. Then, it generates the training, validation and testing sets. Subsequently, the model is trained, tested and its results presented using root mean squared error (RMSE) as the evaluation metric. The user can choose from 5 different ML methods: random forest, support vector machine, and three types of artificial neural networks. Each one of them has a dictionary defining their configuration which can be adapted to suits the user's preferences. Dependencies: Python 3.8.10, pandas 1.2.4.
• *Functions.py* This script contains functions shared among the different scripts.
  Dependencies: Python 3.8.10, pandas 1.2.4, Numpy 1.19.5, Matplotlib 3.4.2, Scikit-Learn 0.24.2, Keras
  2.5.0, TensorFlow 2.5.0.

## 2 Experimental Design, Materials and Methods

**Fig. 1:**
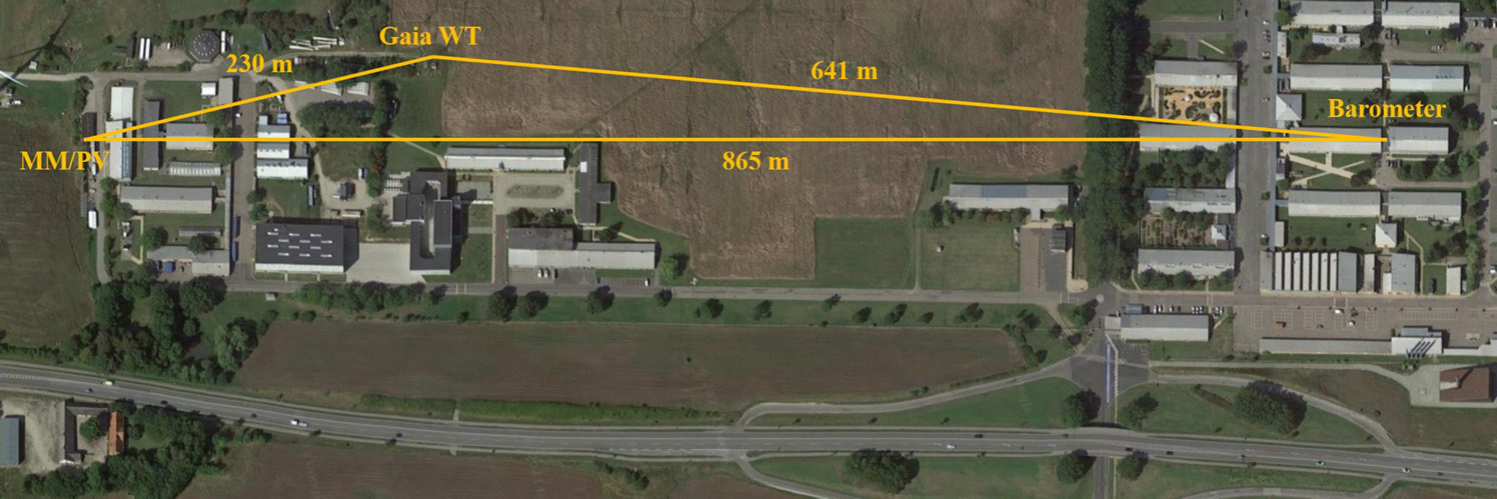
Distances among measuring equipment DTU Risø campus.

The Pyranometers, humidity and Temperature Probe, Wind Vane WZOOP and WindSensor P2546A-OPR Cup Anemometer constitute the meteo-mast (MM). This is located at less than 10 m from the PV array and directly on the roof where the SMA SunnyTripower 10000TL inverter is located at approximately 6 m from the ground. However, the Gaia WT is placed at roughly 230 m, which implies certain displacement between the recorded wind speed at the anemometer and at the turbine. Lastly, the barometer is located 1180 and 970 m from the MM and the WT, respectively. This distances are depicted in Fig. 1. Each device records and samples differently with a minimum of 1 Hz resolution. The recordings are collected in different nodes over the SYSLAB topology. There, they are minimally preprocessed by interpreting the recorded signals into actual SI units and timestamping them. Later, the data from all the nodes is transmitted to a central log.

The data has been retrieved from a DTU server via FTP client, where there is a csv file per day and node. The compiled metrics are distributed into 5 different nodes, hence the raw data has been imported and cleaned from useless strings and other meaningless characters embedded in the original csv files. Then, the data has been time aligned using the timestamps and averaged with a fixed window to obtain the desired resolutions.

## CRediT authorship contribution statement

**Daniel Vazquez Pombo:** Conceptualization, Methodology, Software, Investigation, Data curation, Writing – original draft, Writing – review & editing. **Oliver Gehrke:** Software, Resources, Data curation, Supervision. **Henrik W. Bindner:** Resources, Data curation, Supervision, Project administration, Funding acquisition.

## Declaration of Competing Interest

The authors declare that they have no known competing financial interests or personal relationships that could have appeared to influence the work reported in this paper.

## Data Availability

The SOLETE dataset is available in [1] and the platform containing python scripts in [2].
